# Emerging Characteristics and Properties of Moiré Materials

**DOI:** 10.3390/nano13212881

**Published:** 2023-10-30

**Authors:** Shaofeng Wang, Jizhe Song, Mengtao Sun, Shuo Cao

**Affiliations:** 1 School of Physics, Liaoning University, Shenyang 110036, China; 2 School of Mathematics and Physics, University of Science and Technology Beijing, Beijing 100083, China; d202210426@xs.ustb.edu.cn

**Keywords:** Moiré superlattices, two-dimensional materials, twist

## Abstract

In recent years, scientists have conducted extensive research on Moiré materials and have discovered some compelling properties. The Moiré superlattice allows superconductivity through flat-band and strong correlation effects. The presence of flat bands causes the Moiré material to exhibit topological properties as well. Modulating electronic interactions with magnetic fields in Moiré materials enables the fractional quantum Hall effect. In addition, Moiré materials have ferromagnetic and antiferromagnetic properties. By tuning the interlayer coupling and spin interactions of the Moiré superlattice, different magnetic properties can be achieved. Finally, this review also discusses the applications of Moiré materials in the fields of photocurrent, superconductivity, and thermoelectricity. Overall, Moiré superlattices provide a new dimension in the development of two-dimensional materials.

## 1. Introduction

**Figure 1:**
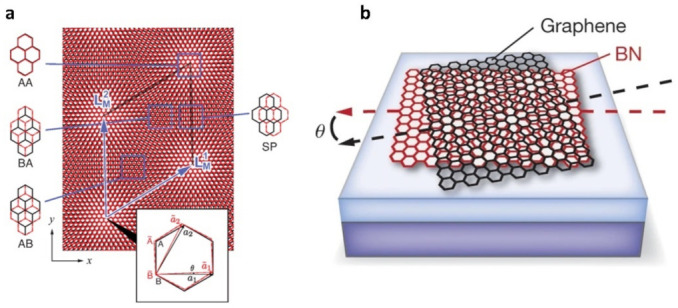
Moiré superlattice. (**a**) Schematic of twisted bilayer graphene [18]. (**b**) Schematic of twisted graphene/BN [16].

Two-dimensional materials have garnered significant interest due to their unique physical properties following the discovery of graphene in 2004 [1]. These materials are stacked together to form different kinds of homo- or heterostructures through van der Waals (vdW) interactions [2,3,4,5,6,7,8]. VdW materials, on the other hand, are those in which van der Waals forces form between layers [5,9,10,11,12]. These interactions lead to the formation of heterojunctions, enriching the performance of 2D materials as various novel physical and chemical properties manifest themselves [13,14,15]. The periodic Moiré pattern is formed when two 2D sheets are stacked and rotated at a specific angle [16]. Moiré superlattice is a heterostructure formed by lattices mismatching or interlayer twisting [17,18,19,20]. As shown in Figure 1, the materials involved can be identical, as seen in twisted bilayer graphene (TBG), or they can be different materials, such as graphene on hexagonal boron nitride (h-BN) [16,18,21,22,23,24]. By artificially rotating one single graphene with another single graphene at a small specific angle, a twisted bilayer graphene is formed, resulting in a low-energy van Hove singularity [25,26,27]. In graphene-h-BN heterostructures, h-BN helps enhance the carrier mobility of graphene by suppressing charge inhomogeneities [28,29]. A periodic superlattice causes the band gap at the Dirac point of graphene to open, [30,31,32] resulting in several unusual quantum phenomena, including the quantum Hall effect and the Hofstadter butterfly pattern. This is a noteworthy advancement in the field [16,33,34]. The atomic and electronic structure of vdW heterostructures is subject to periodic modulation by Moiré superlattices (MSLs), leading to the appearance of several phenomena such as the formation of shear solitons and topological point defects [35,36,37,38], secondary Dirac cones [39,40,41], and Hofstadter butterfly states [33,42,43,44]. In addition, flat bands were discussed back in 2011; such flat energy bands are of interest and may localize the presence of electronic states. Related insulator behavior is found, and unconventional superconductivity is found in a TBG of approximately 1.1° [45,46,47]. Visualization of flat bands by using angle-resolved photoemission spectroscopy with nanoscale resolution (nanoARPES) was reported in 2021 [48,49].

The Moiré superlattice offers new opportunities for the development of two-dimensional materials, providing new dimensions in scientific research in the areas of force, thermal, optical, and electrical fields. This comprehensive review discusses the properties and diverse applications of Moiré materials, encompassing superconductivity, the fractional quantum Hall effect, ferromagnetism, antiferromagnetic, and the topological characteristics of Moiré superlattices. Additionally, it explores the versatile utilization of Moiré materials in photocurrent devices, superconducting quantum interferometry devices, mechanical flexibility, and thermoelectric applications.

## 2. Properties

### 2.1. Superconductivity of Moiré Superlattice

The formation of an electronic flat band can stem from various sources, including topological factors, symmetry considerations, and interactions [50,51]. In the case of graphene layer twisting, the topological mechanism is responsible for the flat band formation. Another reason for the emergence of a nearly flat band may be the spontaneous development of a misfit dislocation array at the interface. The topological aspect of this flat band can also be explained in terms of the pseudo-magnetic field generated by strain [52]. These topologically protected band-touching lines in the graphite spectrum, [53] known as Dirac lines, result in an approximately flat band on the surface of graphite or at the interface. Flat bands offer distinct advantages due to their ability to ensure a high density of states, which enhances the impact of interactions. Consequently, superconductivity induced by electronic correlations becomes apparent at temperatures that are remarkably close to room temperature [54].

**Figure 2:**
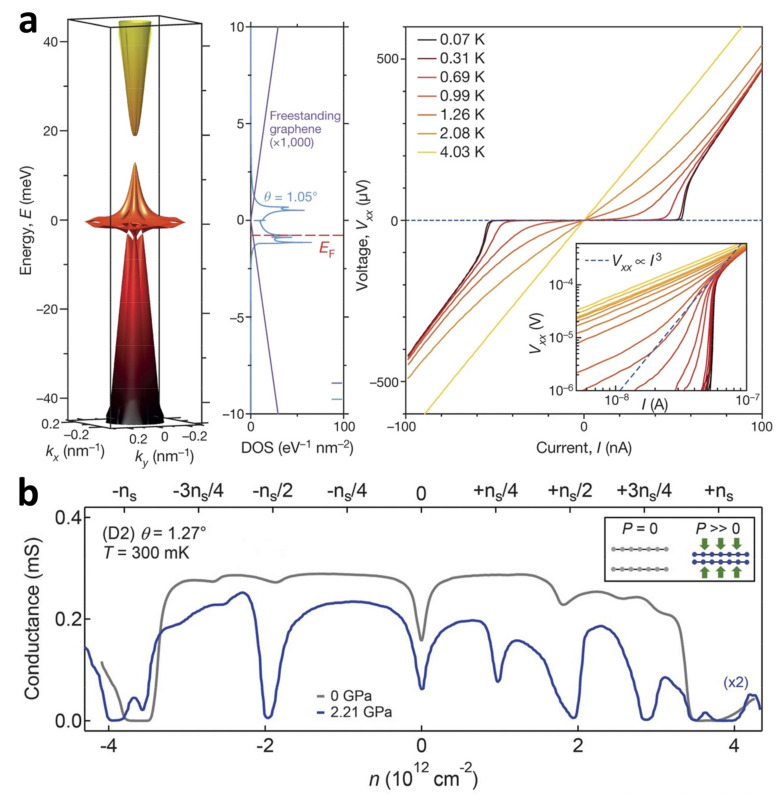
(**a**) TBG (θ = 1.05°) energy band and the corresponding DOS. Measured V_xx_–I curves for devices at different temperatures [46]. (**b**) The conductivity of TBG (θ = 1.27°) is modulated by the pressure modulation results. The inset shows the effect of pressure on the interlayer distance [55].

**Figure 3:**
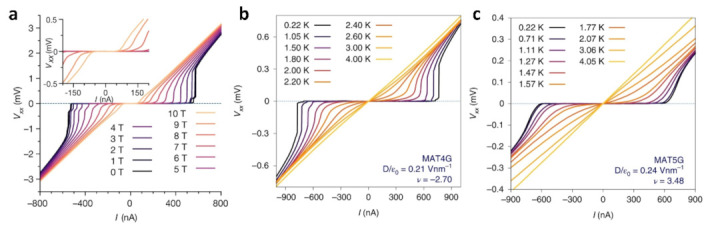
(**a**) V_xx_–I curves for T3G modulated by in-plane magnetic fields [56]. (**b**,**c**) V_xx_–I curves for T4G and T5G at different temperatures [57].

Twisting two monolayer graphene sheets to a specific angle while applying an electric field to control charge density can induce superconductivity at low temperatures. This discovery has ignited global interest in researching twisted-angle graphene systems [46]. In Figure 2a, the device exhibits superconductivity when the Fermi energy of the TBG with θ = 1.05° approaches half of the filling of the lower flat band. The flat-band energy level exhibits energy levels that are over 1000 times greater than the energy levels of two separate, unconnected graphene sheets. This phenomenon occurs as a result of reduced Fermi velocity and increased localization near the magic angle. In Figure 2a, when examining the I–V_xx_ curves of devices with an angle of θ = 1.05° at various temperatures, it is evident that they exhibit the characteristics of a 2D superconductor. Notably, the curve shows a critical current of about 50 nA at temperatures as low as 70 mK. The electrical conductivity plotted against the density of electron holes for a TBG device featuring a twist angle of θ = 1.27° is displayed in Figure 2b. The strong insulating state at 0 GPa appears at ±n_s_ of the fully filled Moiré cell, indicating the existence of an isolated low-energy band. The feeble insulating states are shown near ±n_s_/2 and ±3n_s_/4, and there is no indication of superconductivity. These observations suggest that the low energy band is not significantly involved at this angle. The insulating state becomes apparent at several reasonable fillings of the molar unit cell at 2.21 GPa, most notably at ±n_s_/2 and +3n_s_/4, and even weaker at +n_s_/4. In Figure 3a, the evolution of the V_xx_–I (voltage–current) curve as a function of B_∥_ for twisted three–layer graphene (θ = 1.57°, with adjacent layers sequentially twisted by θ and −θ) shows the robustness of superconductivity (i.e., not the low resistance in the normal state). Despite the fact that the critical current shows a gradual decrease as the parallel magnetic field (B_∥_) increases, it is evident that when B_∥_ reaches 10 T, the V_xx_–I graph still displays a significantly level region for a given DC current bias, suggesting the absence of resistance. However, a sudden increase can be observed in the differential resistance at the critical current. Sharp switching behavior in the V–I curves of twisted four-layer graphene (T4G) and twisted five-layer graphene (T5G) confirms true, robust superconductivity (Figure 3b,c).

**Figure 4:**
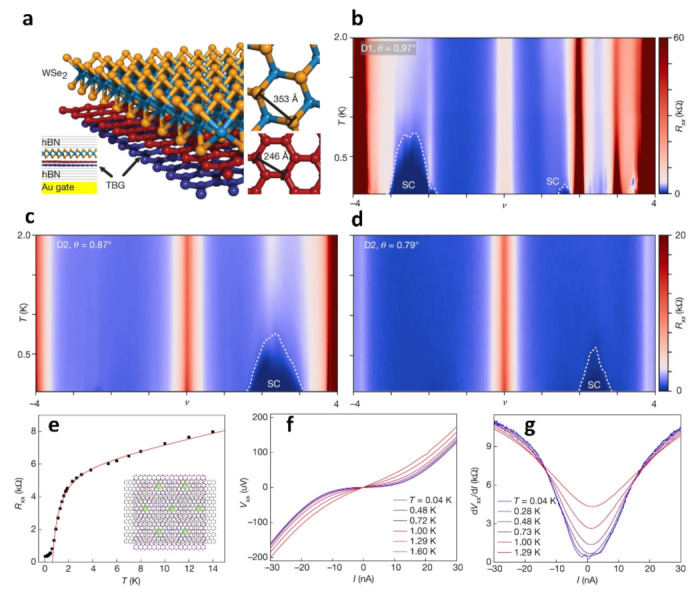
(**a**) Diagram of the structure of TBG-WSe_2_, top view of WSe_2_ and graphene. (**b**–**d**) Relationship between resistance Rxx and temperature and electron density for the three twist angles [58]. (**e**) R_xx_–T curve for trilayer graphene/hBN Moiré superlattice. (**f**) I–V curves of trilayer graphene/hBN at different temperatures. (**g**) dV_xx_/dI–I curves at different temperatures of trilayer graphene/hBN [59].

Figure 4a shows a device made from an hBN-TBG-WSe_2_-hBN van der Waals stack, where the WSe_2_ monolayer is located between the top hBN and TBG instead of the usual hBN-TBG-hBN structure. In Figure 4b–d, the temperature dependence of the resistances of the TBG-WSe_2_ structures (θ = 0.97°, θ = 0.87° and θ = 0.79°) shows superconductivity. The trilayer graphene/hBN Moiré superlattice shows a significant decrease in resistance over a small temperature range (Figure 4e). At the lowest temperature, the I–V curve demonstrates a critical current of less than 10 nA, while at higher temperatures, it shows a near-linear behavior. The differential resistance in Figure 4g highlights a critical current of approximately 10 nA at temperatures below 0.3 K, which develops into normal metallic behavior at temperatures above about 1 K, progressing to normal metal behavior above approximately 1 K.

### 2.2. Fractional Quantum Hall Effect of Moiré Superlattice

At extremely low temperatures, a 2D electron gas exposed to a perpendicular magnetic field undergoes energy quantization into discrete Landau levels. In the presence of a strong magnetic field, the Hall conductance exhibits a quantum plateau as the field strength varies. These plateaus exist near the filling factor of the Landau energy level by an integer or a specific fraction and are known as the integer or fractional quantum Hall effect. The investigation of topological states originated from the experimental observation of fractional quantum Hall effects in 1982, as reported in reference [60].

The fractional quantum Hall effect, as a topological state due to inter-electron interaction, has attracted widespread interest due to its fractionalized quasiparticle excitation. Back in 2013, the quantum Hall effect of graphene was observed, and the fractional quantum Hall effect was discussed [34].

**Figure 5:**
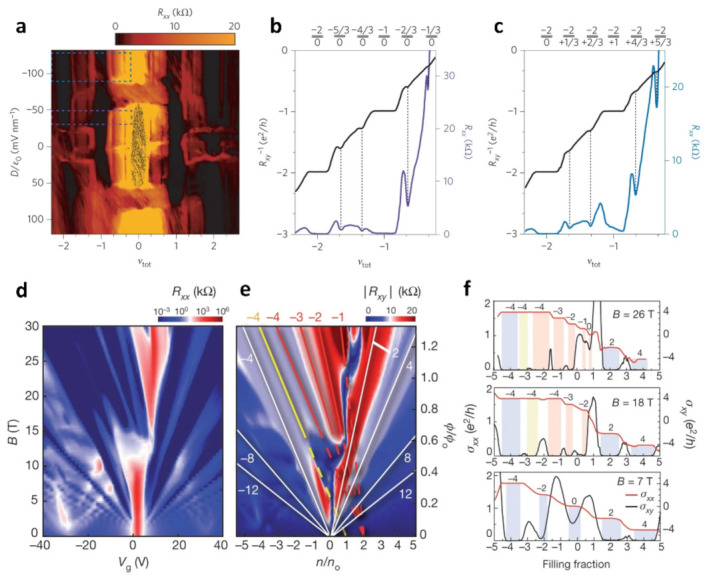
(**a**) R_xx_ at B = 9 T as a function of ν_tot_ and D. (**b**) The R_xx_ and R_xy_ lines in the purple rectangular range in a. The result is a fractional state for the top layer and an insulating state for the bottom layer, respectively. (**c**) The R_xx_ and R_xy_ lines in the blue rectangular range in a. The data correspond to the electron–hole combination. The plotted lines in b and c are averages of the measured quantities over a range of D field values as indicated respectively by the purple and blue rectangles in the colourmap of a. (**d**) Resistance R_xx_ as a function of B(T) and V_g_(V). (**e**) Resistance R_xy_ as a function of ϕ/ϕ_o_ and n/n_o_. (**f**) Longitudinal and transverse Hall conductivity at different magnetic fields. Yellow and red bars indicate correspondence to the similarly coloured anomalous features marked by solid lines in d, e. Blue bars indicate the conventional QHE features.

Figure 5a–c shows the Fractional quantum Hall effect in twisted bilayer graphene [61]. The R_xx_ at B = 9 T shows several significant minima (Figure 5a), while one graphene layer exhibits a ν = 0 insulating state (Figure 5b). A significant fractional quantum Hall effect was found in the electron–hole in the TBG. The detection of fractional quantum Hall states sets the foundation for achieving a fractional quantum spin Hall state, which is an essential component in current plans to create fractional versions of Majorana fermions [62,63,64]. Figure 5d–f shows the anomalous quantum Hall states of twist graphene/h-BN [16]. These are the results of a study on magnetic transport at high magnetic fields. The authors focus on the evolution of R_xx_ and R_xy_ at magnetic fields up to 31 T and plot these quantitative values on images of adjustable experimental gate voltages and magnetic fields, respectively, as well as on dimensionless parameters that appear in the Diophantine equation. They compare their results with those of conventional quantum Hall systems and find that at large magnetic fields, in addition to the usual sequence of bilayer graphene, several additional quantum Hall effect (QHE) states appear, which exhibit minimal values in R_xx_ and plateaus in R_xy_ and exhibit linear trajectories when plotted on the Landau sector diagram. These anomalous QHE states are characterized by integer-valued intercepts s and slopes t and are consistent with a fully developed spectral gap arising from a Hofstadter-type energy spectrum. The authors present conclusive proof that the quantum Hall effect properties related to the Hofstadter spectral gap can be identified by the intercept and slope quantum numbers in the Wannier diagram.

**Figure 6:**
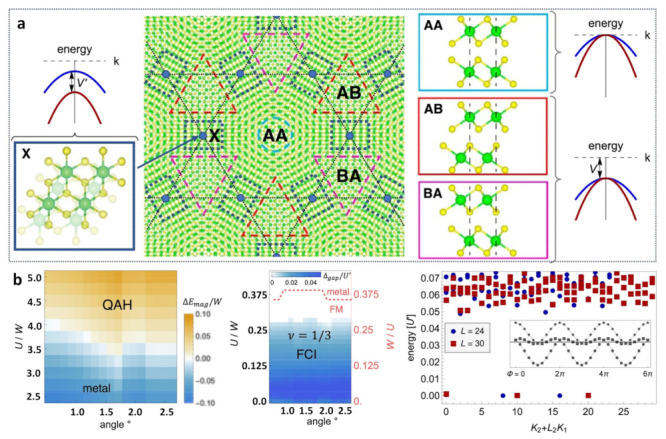
(**a**) Twisted heterostructures of ZrS_2_ and degradation of the electronic state. (**b**) Quantum anomalous Hall effect and fractional Chern insulators [65].

The quantum Hall effect appears not only in twisted bilayer graphene but also in other Moiré materials, such as twisted ZrS_2_ heterostructures. The Moiré superlattice of twisted heterostructures of ZrS_2_ stacks in AA, AB, and BA (Figure 6a) [65]. Neglecting spin-orbit coupling, the valence bands degenerate at Γ in both the AA and AB regions. A very strong anomalous Hall effect appears, as shown in Figure 6b. The difference, however, is that it is not the same as the QHE in the TBG. This phenomenon relies on the intrinsically topologically non-trivial Moiré band structure because of spin-orbit coupling and avoids the simultaneous valley polarization and base effects.

### 2.3. Ferromagnetic of Moiré Superlattice

**Figure 7:**
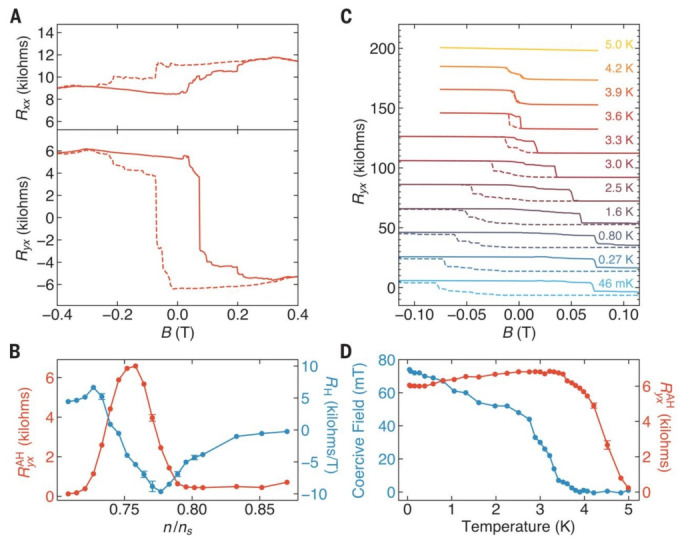
In TBG, (**A**) magnetic field dependent longitudinal resistance R_xx_ and Hall resistance R_yx_. (**B**) AH resistance R_yx_ and R_H_ as a function of n/n_s_. (**C**) Temperature-dependent R_yx_ as a function of B. (**D**) Coercive field and AH resistance R_yx_ as a function of temperature [73].

TBG experiences pronounced electron interactions at a specific angle [66,67,68]. At a particular electron density, magic angle graphene exhibits magnetic properties [69,70,71,72]. Theoretical calculations suggest that this magnetic behavior arises due to the interaction, which lifts spin and valley degeneracy. Typically, the magnetic transport in graphene heterostructures remains unaffected by an applied magnetic field. However, in Figure 7 [73], the magnetic field-dependent resistance in the TBG shows a remanence phenomenon (Figure 7A,B). The transport is hysteretic with respect to an applied out-of-plane magnetic field B (Figure 7A). This phenomenon is important because there are neither transition metals nor heavy elements in the TBG, and the response of the TBG to the magnetic field is related to the effect. The hysteresis loop closes when the temperature rises (Figure 7C,D).

**Figure 8:**
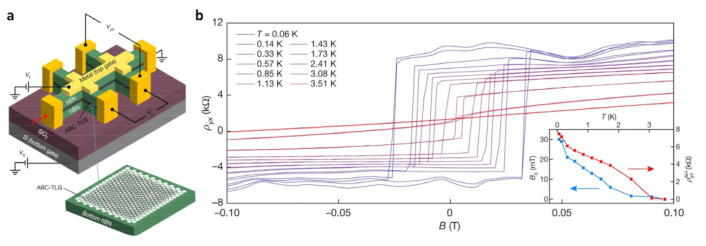
(**a**) Schematic of the ABC-trilayer graphene/hexagonal boron nitride (ABC-TLG/hBN) moiré superlattice Hall bar device. The inset shows that the moiré pattern exists between ABC-TLG and bottom hBN. (**b**) Magnetic-field-dependent ρ_yx_ at different temperatures [79].

The ABC-TLG/hBN heterostructure offers a highly compelling platform for investigating interrelated topological phenomena [74,75,76,77,78]. Figure 8a shows the ABC-trilayer graphene/hexagonal boron nitride (ABC-TLG/hBN) moiré superlattice Hall bar device [79]. In Figure 8b, the graph depicts the Hall resistivity as a function of temperature while subjecting a minor perpendicular magnetic field to a sweeping range of −0.1 T to 0.1 T. Notably, the Hall resistivity exhibits a pronounced anomalous Hall signal accompanied by a robust ferromagnetic hysteresis. The correlated Chern insulator retains its properties even in the absence of a magnetic field, leading to the spontaneous violation of time-reversal symmetry. This state can also give rise to valley-flavor ferromagnetism when filled to one-fourth of its capacity. Notably, ferromagnetic behavior and significant anomalous Hall signals manifest in the Chern insulator state, even when no external magnetic field is applied.

### 2.4. Antiferromagnetic of Twisted Bilayer CrI_3_

**Figure 9:**
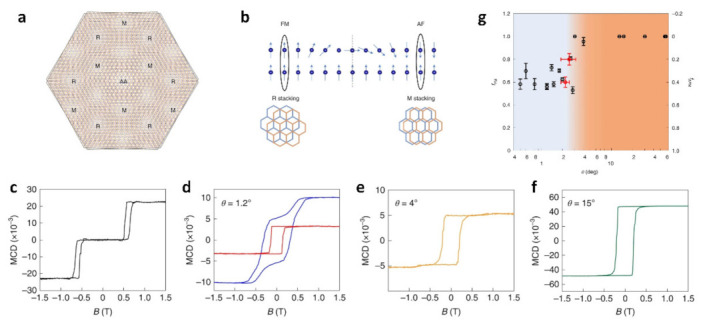
(**a**) Schematic diagram of the twisted bilayer CrI_3_ structure. R, M, and AA are shorthand for these stacking methods, i.e., rhombohedral, monoclinic, and AA stacking. (**b**) The magnetic domain wall appears between R and M. (**c**–**f**) MCD microscopy of twist bilayer CrI_3_, where MCD of bilayer CrI_3_ is shown in a, and the red line in b is the MCD of a monolayer of CrI_3_. Black, blue, yellow and green lines indicate natural bilayer CrI_3_ (a) and twisted bilayer CrI_3_ with torsion angles of 1.2°, 4° and 15°, respectively. (**g**) Twist angle θ dependent AM and FM [82].

In Moiré materials, Moiré superlattices are capable of generating new magnetic properties through interlayer coupling interactions [77,78,79,80,81]. The researchers found that the coexistence of ferromagnetism (FM) and antiferromagnetism (AF) occurs in twisted bilayers of CrI_3_ at small angles [78]. Three types of stacking appear in the twisted bilayer CrI_3_ (Figure 9a) [82]. The occurrence of the magic pattern results in the formation of a magnetic domain boundary separating the R and M regions. (Figure 9b). On the scale of the Moiré length, the competing interactions between interlayer antiferromagnetic and ferromagnetic forces can generate magnetic ground states that are non-trivial, featuring coexisting domains of antiferromagnetic and ferromagnetic properties [83]. At 1.2° ferromagnetism and antiferromagnetism coexist. As θ increases, AF behavior decreases. At 4°, there is small AF behavior; at 15°, there is only FM behavior (Figure 9c–f). Above the critical angle θ_c_ ≈ 3°, only the FM response is observed as the AF-FM coexistence disappears (Figure 9g). However, below θ_c_, we observe that FM, Bc, and FM are all only slightly dependent on the angle.

### 2.5. Topological of Twisted Monolayer-Bilayer Graphene

Understanding the quantum properties in crystalline solids hinges on grasping the fundamental concept of the topological phase of matter [84]. The topological classification hinges on the presence of an energy spectrum gap, with the topological invariant determined by how eigenstates below this gap vary concerning the Bloch wave number [85,86]. Quasicrystalline systems have garnered significant interest due to their topological characteristics. If two atomic layers are superimposed at an arbitrary rotation angle, it typically results in a lack of alignment between the periodic patterns of the individual layers, causing the entire system to exhibit quasiperiodic behavior. Expanding beyond the bilayer scenario, when the relative twist angles form rational ratios, a periodic pattern emerges in the quasimomentum space, resulting in moiré Bloch bands. This occurs even when the system lacks a crystalline lattice structure in real space [87]. One notable characteristic of these 2D twisted quasicrystals is their electronic structure, which can be systematically altered through adjustments in the twist angle or by introducing changes to the lattice structure of individual layers [88].

**Figure 10:**
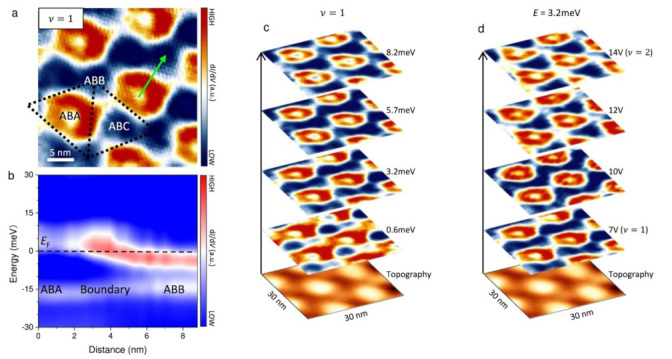
(**a**) Plot of dI/dV at v = 1. (**b**)Variation of dI/dV intensity from ABA to ABB regions along the direction of the arrow in (**a**). (**c**) Variation of dI/dV with energy at ν = 1. (**d**) Plot of dI/dV at different doping for E = 3.2 meV [94].

The lattice misalignment between layers of the vdH heterostructure can lead to the emergence of flat bands, a phenomenon that can be explored for related quantum phenomena [45,46,47]. The potential reduction of spatial symmetry when multi-layered graphene is twisted leads to topological non-trivial in flat bands [89,90,91]. Twisted monolayer-bilayer graphene (tMBG) possesses an inherent non-trivial band topology that distinguishes it from the Chern bands found in TBG [92,93]. The filling state of the conduction flat band (CFB) in tMBG with θ = 1.04° determines the topological properties of the system. The CFB filling is induced within the Moiré lattice, and accordingly, there is a change in the topological properties [94]. As shown in Figure 10a, ABB and ABA structures possess dissimilar topological characteristics in the vicinity of the Fermi energy level. Consequently, the density of states experiences a boost owing to the protection of topological boundaries (Figure 10b) [95,96]. An important point to note is that the radius of the circular structure remains constant regardless of changes in energy or doping (Figure 10c,d). This formation of ring structure originates from topologically protected boundary states. By combining a relevant driven electronic crystal with a ribbon-shaped topological structure, it is possible to create topologically protected states and lattice structures with circular geometry [94].

## 3. Applications of Moiré Superlattices

### 3.1. Photocurrent of TBG Device

**Figure 11:**
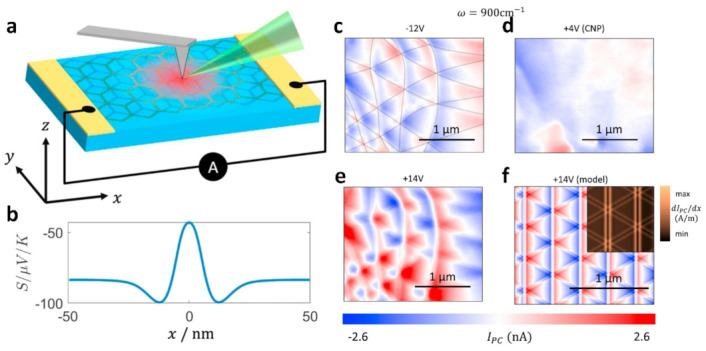
(**a**) Schematic of the scanning photocurrent of a small angle TBG device. (**b**) Seebeck coefficient curves. (**c**–**e**) Illustration of photocurrent at different bias voltages. (**f**) The photocurrent pattern was computed utilizing the Shockley-Ramo formalism [97,109].

A new type of photodetector called scanning nanophotocurrent imaging has the ability to detect and pinpoint changes in the DC transport characteristics of graphene at the nanoscale level [97]. Small angles of TBG change the electronic properties due to interlayer coupling [98,99,100,101]. It is shown that bias modulation opens the band gap of graphene [102,103,104,105]. In TBG devices, researchers concentrate the infrared light onto the metal tip to amplify the electric field beneath it (Figure 11a) [97]. This amplified electric field produces a photocurrent in the local area, which we detect without applying any bias voltage. Photocurrents are produced in graphene as a result of the photoelectric effect (PTE) caused by variations in electron distribution [106,107,108]. The images in Figure 11c–e use red and blue colors to represent positive and negative currents, respectively, and white areas indicate zero current. The photocurrent image displays the intricate structure of the hexagonal lattice, which corresponds to the magnetic domain walls of the relaxed Mohr superlattice in the TBG. The charge neutral point (CNP) is visible when the gate voltage (VG) is +4 V. When comparing the results for gate voltages of −12 V and 14 V, the photocurrent exhibits a similar zigzag pattern for both positive and negative gate voltages, with the only difference being a change in sign. Figure 11f displays the photocurrent patterns acquired through hyperbolic tip calculations, which accurately replicate both the zigzag pattern and intricate details of the domain walls. The results reveal a correlation between the characteristics observed in the photocurrent images and those present in the Seebeck coefficients (Figure 11b). Specifically, the straight lines running along the y-axis and the zero-crossing contours are a result of the magnetic domain walls themselves, while the curved over-zero contour intersects with the magnetic zone wall, leading to interference from the photocurrent in the adjacent zone wall. These findings demonstrate that photocurrent experiments provide direct insight into the nanoscale variations of Seebeck coefficients within the domain walls.

### 3.2. Superconducting Quantum Interference Device (SQUID)

**Figure 12:**
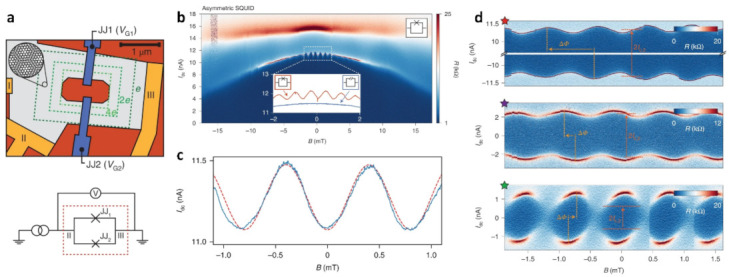
(**a**) Schematic diagram of the SQUI device and circuit equivalents. (**b**) Image of the resistance as a function of I_dc_ and the magnetic field. The illustration shows the trajectory of the critical current line within the oscillation region. (**c**) The image of I_dc_ as a function of the magnetic field showing the current phase relationship (CPR). (**d**) Magnetic interference patterns [114].

Electrostatic doping is a viable method for adjusting numerous relevant material states of the Moiré superlattice [106]. Tunable Josephson junctions have been made possible by the existence of a range of in situ tunable states [110,111,112,113]. Despite the measurement of phase-coherent phenomena, there has been no demonstration of controlling the phase difference between the superconducting condensates thus far. On the basis of the implementation of gate control, a superconducting quantum interference device (SQUID) has been fabricated in TBG [111]. By electrostatic manipulation of the proximity current through the junction in SQUID, its properties can be adjusted (Figure 12a) [114]. At critical currents, oscillations are found, and the superconducting state can be tuned out by electrostatics to suppress the oscillations (Figure 12b). A reduction in the offset 2I_c,2_ between the mean values of the positive and negative switching currents results in a change in symmetry from asymmetry to symmetry (Figure 12d). The current phase relationship (CPR) shows that the applied magnetic field and I_dc_ are sinusoidal (Figure 12c).

### 3.3. Moiré Superlattices Derived from Mechanical Flexibility

**Figure 13:**
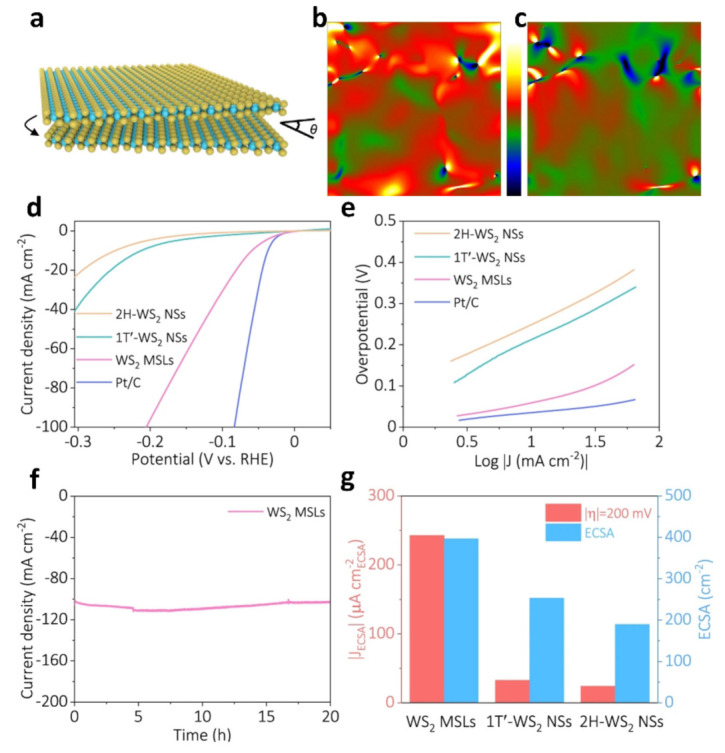
(**a**) WS_2_ Moiré superlattices. (**b**,**c**) Strain distributions of e_xx_ and e_xy_, respectively. (The color from green to dark blue and the color from red to bright yellow represent the compressive strain and tensile strain, respectively). (**d**) Polarization curves of all catalysts. (**e**) The corresponding Tafel curves for catalysts derived from (**d**). (**f**) Continuous HER recorded from synthesized WS_2_ MSLs. (**g**) Comparison of the ECSA and J_ECSA_ [117].

The conventional method of fabricating Moiré superlattices (MSLs) involves stacking materials together, which is highly demanding and requires stringent experimental conditions [115,116]. By applying mechanical strain between WS_2_ layers, a Moiré superlattice with periodic structures is formed. This superlattice structure significantly influences the hydrogen evolution reaction performance of WS_2_ catalysts. WS_2_ Moiré superlattices exhibit excellent catalytic activity and stability, effectively promoting the hydrogen evolution reaction. As illustrated in Figure 13a, the mechanical instability leads to S-W-S layer slippage, which in turn causes the formation of Moiré superlattices [117]. Figure 13b,c demonstrate the successful introduction of strain through topology engineering utilizing mechanical flexibility. Figure 13d reveals that the WS_2_ MSLs exhibit a remarkably low overpotential of only 60 mV vs. RHE at a current density of 10 mA cm^−2^, which is significantly superior to other WS_2_ samples. Figure 13e reveals that the Tafel slope implies that the hydrogen evolution reaction (HER) reaction of WS_2_ MSLs may exhibit a similar mechanism to Volmer–Heyrovsky and is strongly influenced by electrochemical desorption [118,119]. WS_2_ Moiré superlattices exhibit superior hydrogen evolution reaction performance. The results of a 20-h current duration test indicate that the stability of WS_2_ Moiré superlattices remains unaffected during this process. Electrochemically active surface area (ECSA) results reveal that WS_2_ Moiré superlattices possess a higher concentration of enriched activity.

### 3.4. Thermoelectric of Twist Bilayer Borophene

**Figure 14:**
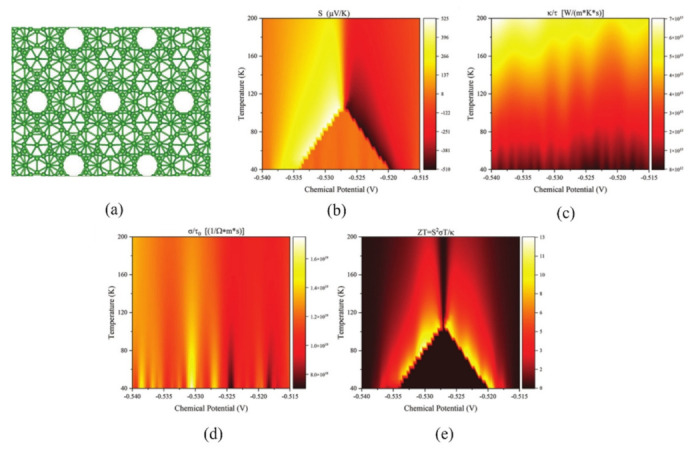
(**a**) $30^{{}^{\circ}}$ twisted α-bilayer borophene, Chemical dependent (**b**) Seebeck coefficient, (**c**) thermal conductivity, (**d**) electrical conductivity, and (**e**) thermoelectric effect [120].

Twisted superlattices made of 2D materials frequently exhibit fascinating properties and can be tuned accordingly. In 2023, Song et al. conducted a theoretical investigation on 30° twisted α-bilayer borophene (TBB) and examined the feasibility of its synthesis (Figure 14) [120]. Twisting the α-bilayer borophene introduces unique interlayer charge-transfer interactions, where electrons are transferred from Ag to TBB. This electron transfer enhances the stability of TBB synthesis on an Ag substrate. Moreover, the act of twisting significantly enhances the thermoelectric performance of bilayer borophene. The inversion of the Seebeck coefficient is a result of the Dirac point splitting near the Γ point. Introducing a twisted superlattice enhances the Seebeck coefficient, increasing it from 300 to 500 μV/K, and raises the optimum temperature to approximately 110 K. Twisting the superlattice creates multiple quantum wells, effectively aligning the high conductivity and large Seebeck coefficient within the same range. The coexistence of large S (Seebeck coefficient) and σ (conductivity) in this region results in an increase in the figure of merit (ZT), demonstrating a notable correlation between the structure and properties of the material.

## 4. Conclusions

Based on the above discussions, Moiré superlattices have been extensively explored for various material properties and application research, including superconductivity, ferromagnetism, antiferromagnetism, topological properties, quantum Hall effect, optoelectronic applications, and so on.

In the field of superconductivity, Moiré superlattices provide a new approach to studying superconducting materials and open up new avenues for superconducting applications. The mechanisms of superconductivity, including critical currents and temperature ranges, need to be further understood. The role of electron–electron interactions, lattice effects, and other factors in promoting superconductivity also needs to be replicated. Moiré superlattices with ferromagnetic, antiferromagnetic, and topological properties also provide new platforms for the study of these materials. The stability of these magnetic behaviors is a key point that cannot be ignored. It is necessary to understand the relationship between twist angles, topological properties, and potential applications, offering the possibility of creating topologically protected states for various electronic and quantum applications. The study of the quantum Hall effect can also be conducted through Moiré superlattices, which is of great significance for developing new electronic devices.

In addition, Moiré superlattices have also been applied in the field of optoelectronics, such as photodetectors, solar cells, opto-control devices, etc. The realization of these applications requires in-depth research on the optoelectronic properties and performance of Moiré superlattices, as well as the development of efficient and controllable preparation methods.

Despite the progress that has been made in various fields using Moiré superlattices, there are still many unresolved issues and challenges, such as how to precisely control the structure and properties of Moiré superlattices, how to improve their preparation efficiency and scalability, and so on. With the continuous advancement of technology and methods, research and applications of Moiré superlattices are expected to continue to develop and provide new solutions for scientific and engineering problems in various fields.
